# Picophytoplankton prevail year‐round in the Elbe estuary

**DOI:** 10.1002/pei3.70014

**Published:** 2024-10-27

**Authors:** Nele Martens, Johanna Biederbick, C.‐Elisa Schaum

**Affiliations:** ^1^ Institute of Marine Ecosystem and Fishery Science Hamburg Germany; ^2^ Center for Earth System Research and Sustainability Hamburg Germany

**Keywords:** picocyanobacteria, picoeukaryotes, salinity, temperature, turbidity

## Abstract

Picophytoplankton are important primary producers, but not always adequately recognized, for example, due to methodological limitations. In this study, we combined flow cytometry and metabarcoding to investigate seasonal and spatial patterns of picophytoplankton abundance and community composition in the Elbe estuary. Due to the mixing of freshwater and seawater and the tidal currents this ecosystem is characterized by typical estuarine features such as salinity gradients and high turbidity. Picophytoplankton (mostly picoeukaryotes such as *Mychonastes* and *Minidiscus*) contributed on average 70% (SD = 14%) to the total phytoplankton counts. In summer picocyanobacteria (e.g., *Synechococcus*) played a more significant role. The contributions of picophytoplankton to the total phytoplankton were particularly high from summer to winter as well as in the mid estuary. However, at salinities of around 10 PSU in the mixing area of freshwater and seawater, the proportion of picophytoplankton was comparably low (average 49%, SD = 13%). Our results indicate that picophytoplankton prevail in the Elbe estuary year‐round with respect to cell counts. Picophytoplankton could occupy important niche positions to maintain primary production under extreme conditions where larger phytoplankton might struggle (e.g., at high or low temperature, high turbidity, and in areas with high grazing pressure) and also benefit from high nutrient availability here. However, we did not find evidence that they played a particularly significant role at the salinity interface. Our study highlights the importance of including picophytoplankton when assessing estuarine phytoplankton as has been suggested for other ecosystems such as oceans.

## 1 INTRODUCTION

Picophytoplankton (<2–3 μm) are important primary producers in aquatic ecosystems from oligotrophic to eutrophic habitats (Coello‐Camba & Agustí, 2021; Moreira‐Turcq et al., 2001; Purcell‐Meyerink et al., 2017; Takasu et al., 2023; Zhang et al., 2015). These tiny organisms fulfill crucial ecological functions, for example, as food for nauplii larvae and filter feeders (Bemal & Anil, 2019; Richard et al., 2022) and in carbon export (Basu & Mackey, 2018; Puigcorbé et al., 2015). The small size of picophytoplankton allows them to occupy specific ecological niches, for example, due to the high surface to volume ratio which might facilitate the uptake of required nutrients, and slow sinking velocity that can keep them in the euphotic zone (Massana, 2011; Raven, 1998). Short generation times and large standing genetic variation give picophytoplankton a comparatively high evolutionary potential (e.g., Barton et al., 2020; Benner et al., 2020; Schaum et al., 2016). Picophytoplankton are more than likely to prevail in changing environments (see, e.g., Benner et al., 2020; Flombaum & Martiny, 2021; Tan et al., 2022). Some picophytoplankton have been shown to appear under extreme conditions, for example, at high or varying salinity, turbidity, and temperature (Belkinova et al., 2021; Somogyi et al., 2022).

Extreme living conditions are common across ecosystems, including estuaries. Estuaries are the interfaces between the freshwater and marine world and characterized by gradients and tidal‐induced variation of environmental forcing (e.g., salinity, turbidity), and picophytoplankton can be an important group here (Moreira‐Turcq et al., 2001; Paerl et al., 2020; Purcell‐Meyerink et al., 2017; Sathicq et al., 2020). However, due to their small size picophytoplankton are still often not adequately recognized. This is largely due to difficulties in detecting and identifying these small‐celled organisms with light microscopy (Bergkemper & Weisse, 2018). Moreover it has been shown that picoeukaryotes cannot be thoroughly preserved with common fixation techniques, and abundances might decline with storage time (Nogueira et al., 2023). Here, we applied flow cytometry and metabarcoding (the latter partially from Martens, Russnak, et al., 2024) to (1) investigate spatial and seasonal patterns in picophytoplankton abundance and composition in the Elbe estuary, (2) identify dominant taxa, and (3) assess under which conditions (with respect to abiotic factors) picophytoplankton and different players within (e.g., picocyanobacteria) might be particularly dominant.

## 2 MATERIALS AND METHODS

**FIGURE 1:**
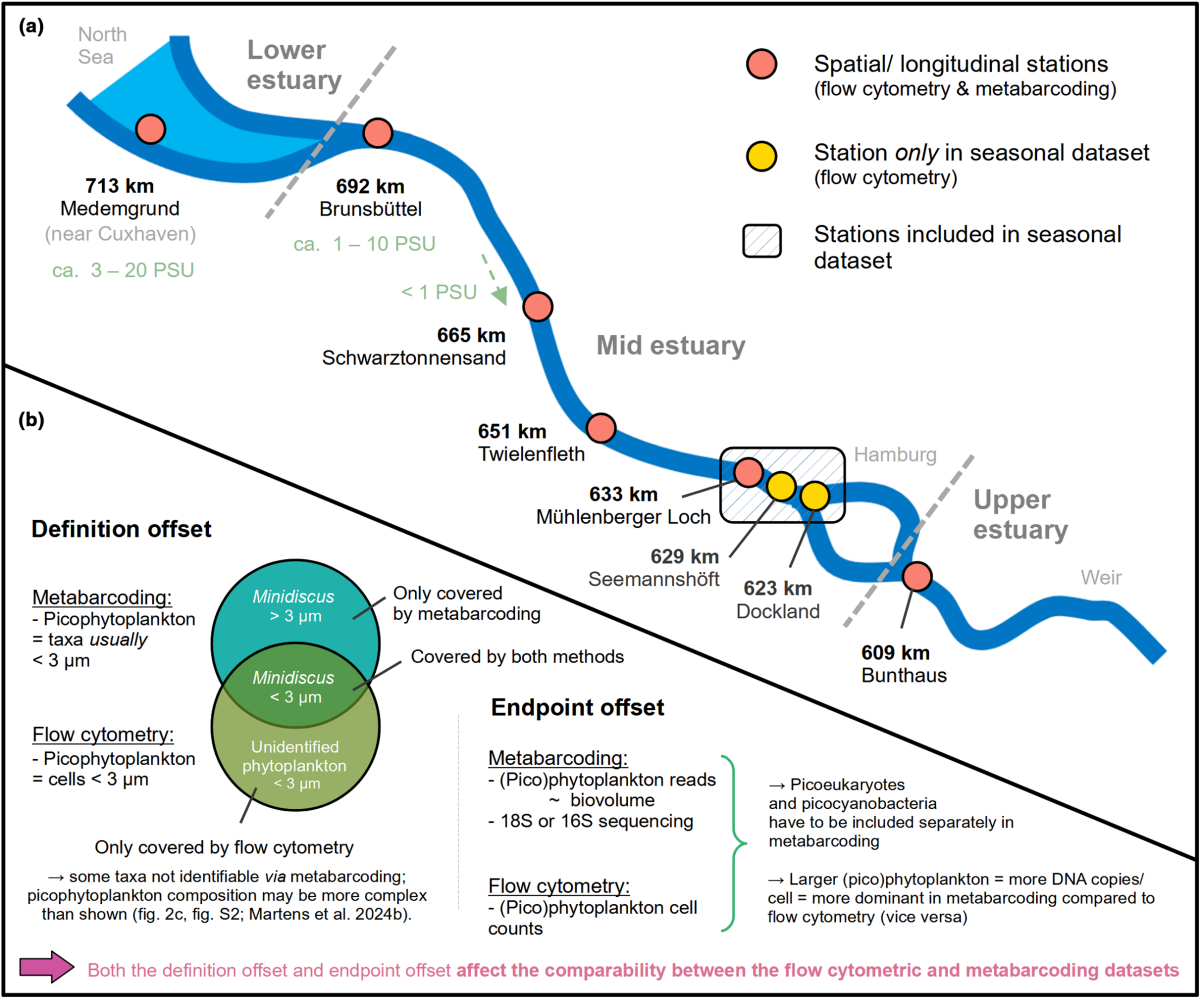
Study area (Elbe estuary) and sampling stations of the seasonal and spatial dataset (a) and schematic overview of the offset between the measured endpoint and definition of picophytoplankton by the different methods (b). In (a) “km” metric indicates the approximate distance from the spring of the Elbe River in the Czech Republic (stream km). In (b), the text within the circles provides examples.

**FIGURE 2:**
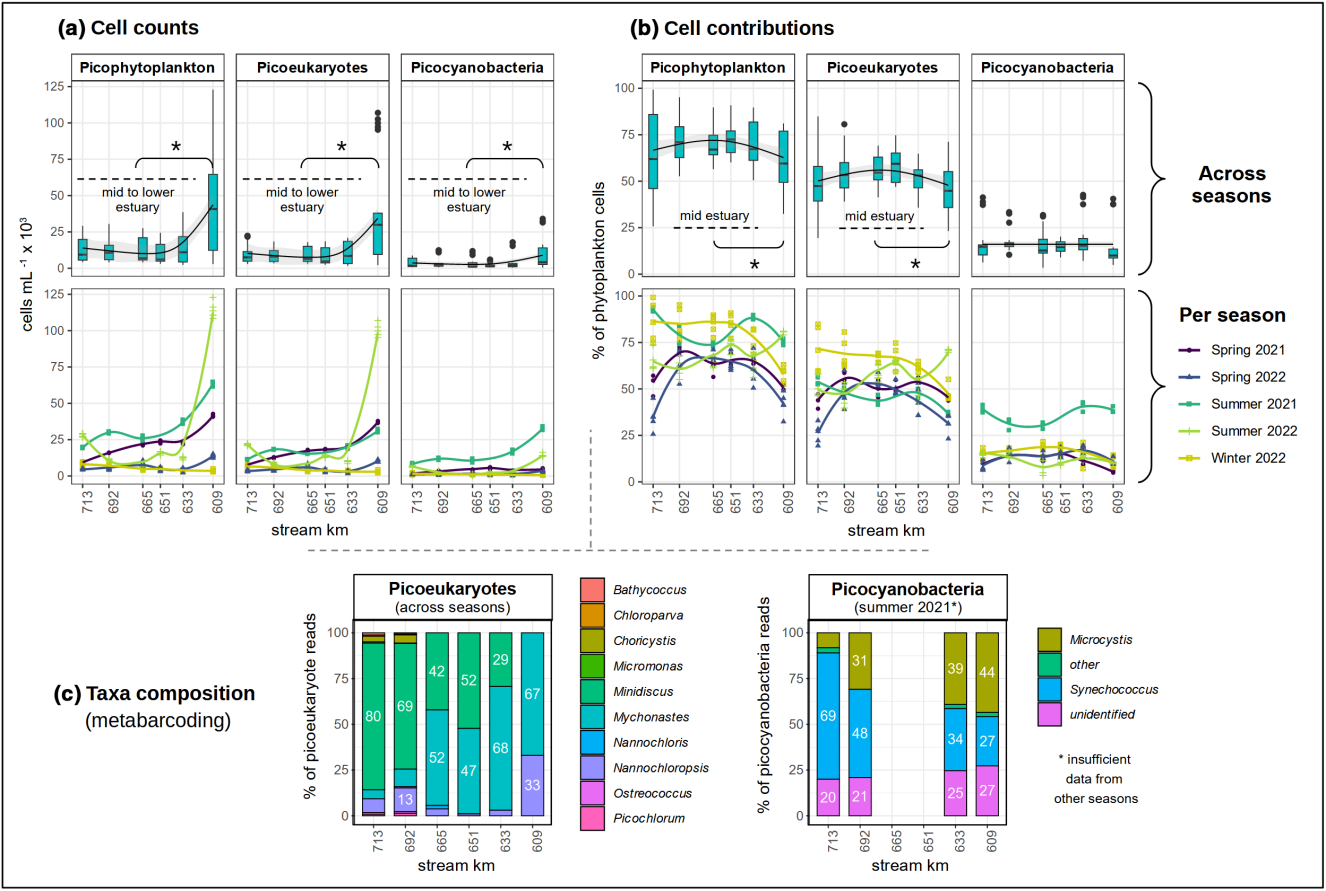
Spatial distribution of different picophytoplankton groups along different stations (stream km). In (a) and (b), the top and bottom rows show the same data, but in the top row these are shown across seasons (regression lines = method “gam” with chosen k; see also Table S3) and in the bottom row they are shown per season (regression line = method “loess” for visual support). In the top row of (a) and (b) we additionally show where values were significantly different (*p* ≤ .05, indicated by *) between the upper estuary (609 km) and the mid estuary (633–692 km) respectively mid to lower estuary (633–713 km) according to an ANOVA and Tukey test (see also Table S2). Data in (c) is partially obtained from a former study (Martens, Russnak, et al., 2024). For clarity, labels are shown for values ≥10% only. Note that metabarcoding was not carried out at 651–665 km in 2021; hence, the averaged composition of picoeukaryotes at these stations across seasons in (c) does only cover data from 2022. Beyond, it should be considered that metabarcoding includes phytoplankton that could not be identified to genus level and hence does not appear in (c) as they cannot be assigned to the size group of picophytoplankton. Metabarcoding data per season can be found in the supplementary data (Figure S2). All data are shown in Table S5.

**FIGURE 3:**
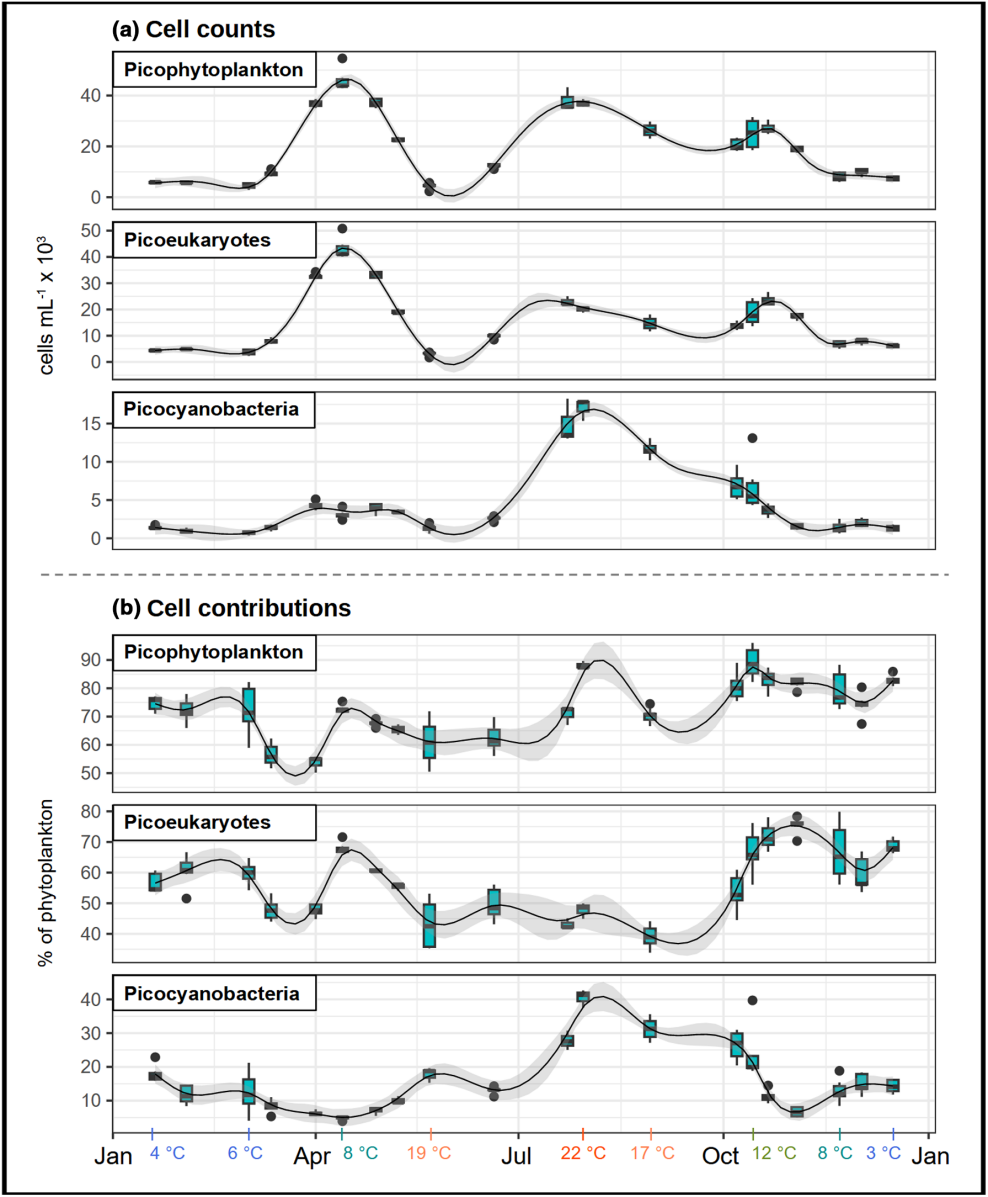
Seasonal distribution of different picophytoplankton groups in the area around the city center of Hamburg (approx. 623–633 km). Horizontal scales show the sampling date independent of the year, that is, day of the month. Data were merged when sampling was carried out <5 days apart. Regression lines were added with geom_smooth() from ggplot2 and the method “gam” with chosen *k* values (see also Table S3). On the bottom we show the temperatures at certain time points (see further details in Figure 4a). All data are shown in Table S5.

The Elbe estuary is located in the North of Germany, passing through the city of Hamburg, and enters the North Sea at Cuxhaven (Figure 1a). As one of Europe's largest estuaries it is an important natural habitat and supplies the human population with essential ecosystem services (e.g., via port of Hamburg, recreation areas). The Elbe estuary has been experiencing intense anthropogenic pressure for centuries, and further changes such as global warming or deepening of shipping channels might have additional impacts on the ecosystem functioning (see, e.g., Van Maren et al., 2015). The tidal estuarine area is separated from the Elbe River by a weir at 586 km distance from the river source. A total of 50 surface water samples (ca. 0–2 m depth) were taken from seven stations along the Elbe estuary during different sampling campaigns (Figure 1a and Table S1). Samples were taken aboard the research vessel *Ludwig Prandtl*, the fishing vessel *Ostetal*, and from two different piers (Dockland, Seemannshöft) in Hamburg. Further details about sampling in the different sampling campaigns—for example, sampling method and sample volume—are given in the supplementary data (Table S1). Twenty‐five samples were taken around the city of Hamburg (approx. 623–633 km) and used as a seasonal dataset (Figure 3), and 29 samples from longitudinal sampling of six stations (609–713 km) covering three different seasons (spring and summer each 2021 and 2022 as well as winter 2022) were used as a spatial dataset (Figure 2 and Figure S2).

Of each sample, 3–5 technical replicates à 20 μL were analyzed using flow cytometry (BD accuri C6 plus) with a flow rate of 66 μL min^−1^ and regular cleaning and mixing between the samples. Phytoplankton cells could be distinguished from other suspended matter by their cytometric properties (e.g., fluorescence, size) which were also used to identify different groups of phytoplankton (see, e.g., Ning et al., 2021; Read et al., 2014; Thyssen et al., 2022; and supplementary material Figure S1). Picophytoplankton in the included samples from the Elbe estuary could be divided into two major groups: picoeukaryotes and picocyanobacteria. Picocyanobacteria differed from picoeukaryotes in their fluorescence properties. This group had a higher phycocyanin‐ and lower chlorophyll‐fluorescence (Figure S1). Notably, some larger cells might be excluded from our analysis due to detection limits and low sample volume. However, we know from former data (see, e.g., Martens, Russnak, et al., 2024; NLWKN, 2023) that taxa <40 μm (e.g., *Stephanodiscus*, *Cyclotella*) are dominant in most seasons and areas of the Elbe estuary.

For the spatial dataset, 16S rRNA metabarcoding and 18S rRNA metabarcoding from another study (see further information in Martens, Russnak, et al., 2024) were included to add information about picophytoplankton taxa in the Elbe estuary (Figure 2c and Figure S2). Samples for 16S rRNA sequencing were processed in the same way as shown for the 18S data (Martens, Russnak, et al., 2024); however, reads were assigned using the BLAST database (carried out by biome‐id Dres Barco & Knebelsberger GbR). In both datasets, we selected taxa that are in general considered picophytoplankton (e.g., *Synechococcus*, *Choricystis*, *Mychonastes*, *Minidiscus*). These are taxa that are *usually* <3 μm; however, this might not always apply for every species, morphotype, and cell within a population. We kept colony‐forming taxa that might appear solitary where single cells *can* be <3 μm (e.g., *Microcystis*)—as well as unidentified cyanobacteria—in the dataset as they might add to the picocyanobacteria counts in the cytometry data. Note that the definition of picophytoplankton in the metabarcoding data is based on taxa identity and their *usual* size ranges, while in flow cytometry the definition is exclusively based on the *actual* cell size (<3 μm) (Figure 1b). Furthermore, while in flow cytometry we detect abundance, metabarcoding results are rather correlated with biovolume. This is due to the size dependence of DNA copies per cell (Godhe et al., 2008), and as a result, larger (picophytoplankton) taxa might appear more dominant in metabarcoding compared to flow cytometric data without being more abundant in terms of cell counts. Consequently, what is included in “picophytoplankton” and how dominant it is can to some extent differ between the methods (see Figure 1b for further details). In addition, missing or insufficient data in metabarcoding (e.g., due to unidentifiable taxa, no sampling at 651–665 km in 2021, and low read numbers) make the comparison with flow cytometry rather difficult. For instance, we excluded data from samples with less than 100 picocyanobacteria, respectively picoeukaryotes reads in metabarcoding (Figure S2). The number of picophytoplankton reads per sample varied from 147 to 6108 (average 1733) in the 18S dataset and 113–5692 (average 2155) in the 16S dataset.

Data were processed in R (version 4.1.3), including the packages tidyverse (version 1.3.2), ggplot2 (version 3.4.0), lubridate (version 1.9.2), scales (version 1.2.1), and MuMIn (version 1.47.5). We also used LibreOffice Draw (version 7.1.2.2) for overview figures and the addition of text notes and chatGPT (GPT‐4) to streamline R code and to check the finalized manuscript for common grammatical and typographical errors. For spatial analyses, we obtained potentially interesting patterns from the figures showing cell counts and contributions of picophytoplankton groups along stations (Figure 2a,b) and then carried out an ANOVA aov() and Tukey test TukeyHSD() from the package stats (version 4.3.1) to assess whether the observed patterns were significant. To do so, we partially clustered different stations together, for example, those in the mid estuary (see also Figures 1 and 2a,b and Table S2). In Figures 2a,b and 3 we used GAMs for curve fitting with geom_smooth() from ggplot2 and the formula y ~ s(*x*, bs = “cr”, *k*). The *k* value describes the number of knots. Knots are the boundaries of the piecewise splines that define the GAM. They describe how often the fitted curve can change, for example, in terms of direction and steepness. The higher the *k* value, the more complex the GAM. The *k* values were determined based on the lowest AIC as obtained from uGamm() from the package MuMIn and AIC() from stats (see Table S3).

**FIGURE 4:**
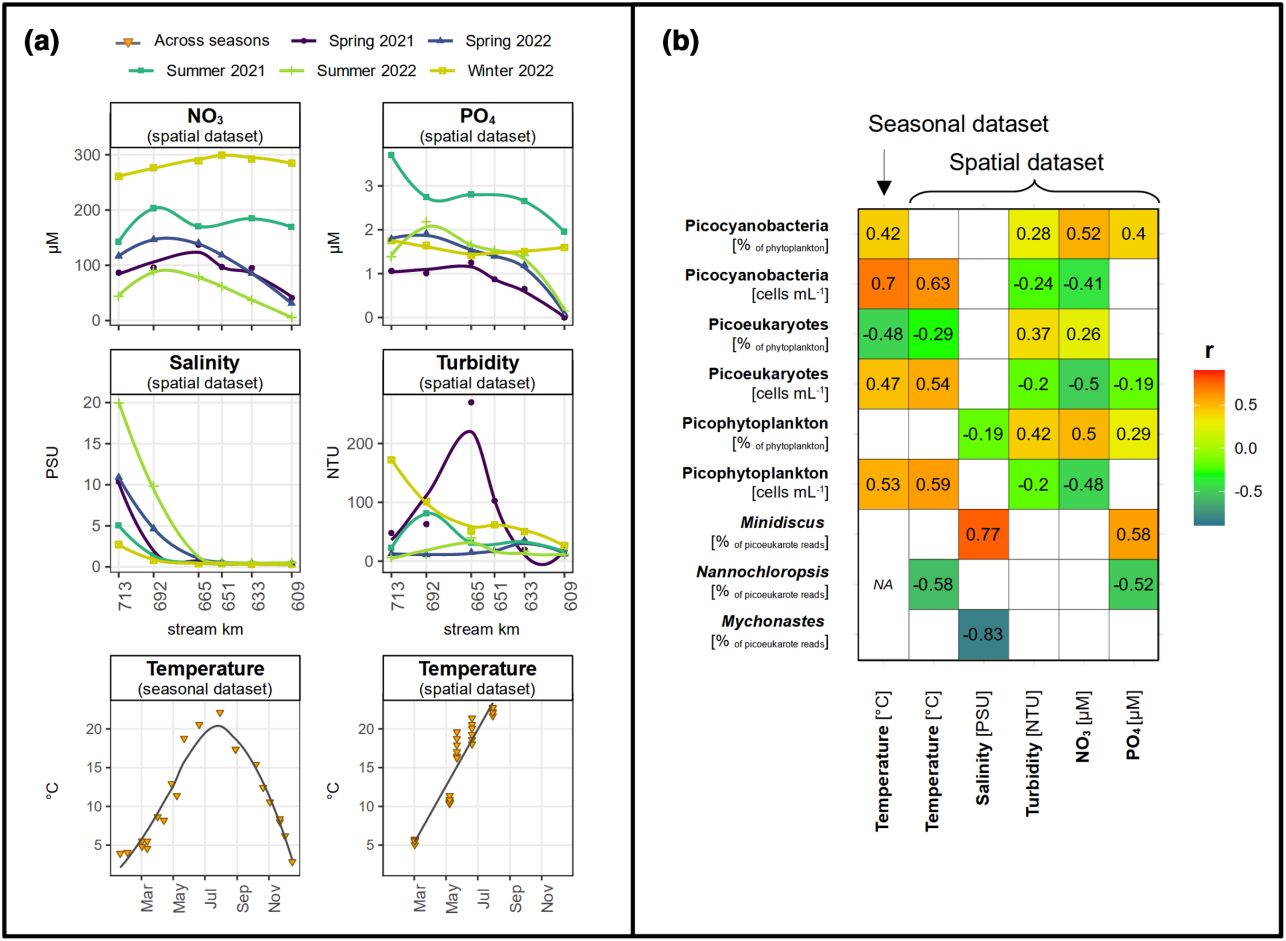
Abiotic conditions (a) and correlation of different picophytoplankton groups with the abiotic conditions (b) in the spatial and seasonal dataset. Regression lines in (a) were added with geom_smooth() from ggplot2 and the method “loess” and “lm” as visual support. Note that one missing value of turbidity on July 29, 2021 (summer 2021) at 609 km was replaced by a value from July 26, 2021, at 609 km. Numbers and color scheme in (b) show the correlation coefficient r calculated with Spearman rank correlation for *p* ≤ .05 (see also Table S4). 16S data were not included due to the low number of data points (see methods).

To set the phytoplankton distributions into context with the environmental conditions, additional abiotic parameters (water temperature, salinity, turbidity, PO_4_, and NO_3_; see also Figure 4 and Table S4) were obtained during the sampling campaigns. Temperature, salinity, and turbidity were measured with a FerryBox (Petersen et al., 2011) during the sampling cruises. For samples taken from the pier in Hamburg (i.e., at Seemannshöft or Dockland, see Table S1, Figure 1a), temperature and salinity were measured with a portable handheld sensor (Hanna Instruments, Vöhringen, Germany; model number HI98494). Nutrient analysis was carried out by Helmholtz‐Zentrum hereon. Samples for NO_3_ and PO_4_ analysis were collected through the flow‐through pump system of the FerryBox and filtered through combusted, pre‐weighted GF/F filters (4 h, 450°C), and stored in acid‐washed (10% HCl) PE bottles at −20°C. Three replicates were analyzed using an automated continuous flow system (AA3, Seal Analytical, Germany) and standard colorimetric techniques (Hansen and Koroleff 2007), and the mean values were included in this study. A Spearman rank correlation with the function rcorr() from the package Hmisc() (version 5.1–0) was applied to draw conclusions about the relationship of picophytoplankton groups with abiotic parameters (Figure 4b and Table S4). Additionally, turbidity data were obtained from the FGG database (FGG Elbe, 2024) to compare these qualitatively with the seasonal dataset, where turbidity was not measured.

## 3 RESULTS

Across seasons and stations, flow cytometry detected between 2.3 × 10^3^ and 123 × 10^3^ picophytoplankton cells mL^−1^ in the samples from the Elbe estuary. On average 70% (SD = 14%) and up to 99% of the detected phytoplankton cells per sample were <3 μm. Picoeukaryotes were by far the most dominant group with an average contribution of 77% (SD = 11%) to the picophytoplankton cell counts, while picocyanobacteria played a role in summer (up to 53%).

Across seasons, picophytoplankton, picoeukaryotes, and picocyanobacteria were overall significantly more abundant at the uppermost station (609 km) than in the area further downstream (633–713 km) (Figure 2a, ANOVA/Tukey: *p* = 6.0 × 10^−11^, *p* = 2.1 × 10^−10^ and *p* = 3.7 × 10^−6^, respectively; see also Table S2). Contributions of picoeukaryotes to the phytoplankton cell counts were significantly higher in the mid estuary (633–692 km) compared to the upper station (609 km) (Figure 2b, ANOVA/Tukey: *p* = .009; Table S2), while they showed no significant differences between the mid and lower, as well as the upper and lower stations (Figure 2b and Table S2). As the picophytoplankton fraction was largely represented by picoeukaryotes, those patterns hold for the contributions of picophytoplankton to the phytoplankton cell counts as a whole (Figure 2b, ANOVA/Tukey: *p* = .031 for comparison of the mid (633–692 km) and upper area (609 km); Table S2). In contrast, contributions of picocyanobacteria to the picophytoplankton did not express a distinct pattern along space across seasons (Figure 2b).

*Minidiscus* and *Mychonastes* were the most dominant picoeukaryote taxa across seasons based on 18S rRNA reads (Figure 2c). Therein, *Mychonastes* was more dominant in the upper to mid reaches of the estuary (approx. 609–665 km), and *Minidiscus* in the mid to lower area (approx. 651–713 km). *Nannochloropsis* was prominent at 609 km in early May (spring 2021) and at 692–713 km in February (winter 2022) (Figure S2a). Here *Choricystis* also played a role (contributions up to approx. 20%). Other picoeukaryotes such as *Bathycoccus* and *Picochlorum* were minor contributors to the 18S picophytoplankton reads. Results from 16S sequencing (Figure 2c) show that *Synechococcus* and *Microcystis* might be the most relevant contributors to picocyanobacteria in summer 2021, where picocyanobacteria were particularly dominant (up to approx. 43% of the phytoplankton cells; see also Figure 2b). Here *Microcystis* was more dominant at the upper stations (609–633 km) and *Synechococcus* at the lower stations (692–713 km) (Figure 2c). Notably there is some degree of uncertainty to what extent *Microcystis* would fall into the size range of picophytoplankton, due to colony formation and cell size. It is likely that *Synechococcus* reached significantly higher proportions among the cells <3 μm than suggested in Figure 2c. Minor contributors to the picocyanobacteria reads were, for example, *Prochlorococcus* and *Cyanobium* (“other” in Figure 2c).

In our seasonal dataset from downstream of the city center of Hamburg (623–633 km), the abundances and contributions of the different picophytoplankton groups expressed distinct patterns along the sampling dates. The complexity is reflected in the high *k* values (15–20) of the fitted GAMs (Figure 3 and Table S3). Picophytoplankton expressed seasonal peaks in spring, summer, and fall, largely due to the respective peaks of picoeukaryotes during these seasons and high picocyanobacteria abundances around July to August with elevated abundances extending into October (Figure 3a). Picophytoplankton contributions to the total phytoplankton were highest in a single sample from the temperature peak in summer (Figure 3b), largely due to picocyanobacteria, and across different samples in fall, which is due to low abundance of larger‐celled taxa combined with the fall peak of picoeukaryotes and the remains of the fading summer bloom of picocyanobacteria (Figure 3a,b). In contrast, picophytoplankton were less dominant within the phytoplankton communities in spring (Figure 3b) due to taxa >3 μm blooming in parallel. Seasonal effects could also be observed in the spatial dataset as longitudinal data were obtained from different seasons (winter, spring, and summer). Here, the highest absolute abundances of picophytoplankton were observed in summer 2022 (Figure 2a). Contributions of picophytoplankton to the total phytoplankton counts were overall highest in summer 2021 and in winter 2022 mostly due to picocyanobacteria as well as picoeukaryotes and low abundance of larger‐celled phytoplankton, respectively (Figure 2b).

Abiotic conditions varied along seasons and stations (Figure 4a). Temperature was highest in July (up to 23°C in the spatial dataset at 665 km) and low in winter (down to 3°C). Turbidity, salinity, NO_3_, and PO_4_ expressed spatial and seasonal patterns. Salinity was enhanced at the lowermost stations (692–713 km) and highest in summer 2022 and spring of both years. Compared to other seasons, turbidity was enhanced in winter 2022 and spring 2021, NO_3_ in winter 2022 and summer 2021, and PO_4_ in summer 2021. Note that we lack information about turbidity (as well as NO_3_ and PO_4_) from fall, as this season was not included during the longitudinal sampling campaigns where these parameters were measured. However, we know from further database data that turbidity was also enhanced in fall 2021 (Figure S3; FGG Elbe, 2024). Turbidity and PO_4_ and NO_3_ concentrations were overall enhanced downstream of 609 km (Figure 4a).

Picophytoplankton abundance was positively correlated with temperature (Figure 4b *r* = .59 and .53 for the spatial and seasonal dataset, *p* < .001 each). This was a result of high abundances of both groups in summer—for picoeukaryotes specifically in the spatial dataset in summer 2022 (Figure 2a), and for picocyanobacteria in general (Figures 2a and 3a)—and low abundance of both groups in winter (Figures 2a and 3a). Relative contributions of picoeukaryotes to the phytoplankton were negatively correlated with temperature (Figure 4b *r* = −.29 and −.48 for the spatial and seasonal dataset, *p* < .001 each). Overall this pattern arises from relatively high picoeukaryote contributions to the phytoplankton in fall and winter where phytoplankton abundance was generally low and enhanced picocyanobacteria contributions to the phytoplankton in summer (Figures 2b and 3b). Picophytoplankton contributions to the phytoplankton cell counts were negatively correlated with salinity (Figure 4b *r* = −.19, *p* = .040), largely due to low contributions at around 10 PSU at 713 km in spring 2021 and 2022 (Figures 2b and 4a). Cell counts of picophytoplankton were negatively correlated with turbidity and NO_3_ (Figure 4b, turbidity: *r* = −.20, *p* = .029, NO_3_: *r* = −.48, *p* < .001) due to their high absolute abundance at 609 km—especially in summer—where turbidity and NO_3_ concentrations were rather low (Figures 2a and 4a). In contrast, relative contributions of picophytoplankton to the phytoplankton were positively correlated with these parameters and additionally with PO_4_ (Figure 4b, turbidity: *r* = .42, *p* < .001, NO_3_: *r* = .50, *p* < .001, PO_4_: *r* = .29, *p* = .001). This relationship with PO_4_, NO_3_, and turbidity is affected by the higher proportions of small cells in the mid to lower estuary, where these parameters achieved overall higher values compared to 609 km and by the seasonal importance of picocyanobacteria in summer 2021 (at high PO_4_) and picoeukaryotes in winter (at high turbidity and NO_3_) (Figures 2b and 4a). Due to enhanced contributions to the picoeukaryotes reads from winter to spring compared to the other seasons (Figure 2c), *Nannochloropsis* was negatively correlated with temperature (Figure 4b *r* = −.58, *p* = .010). The negative relationship with PO_4_ (Figure 4b, *r* = −.52, *p* = .022) can be mainly explained by the high contributions of this taxon at 609 km in spring 2021, where PO_4_ was particularly low (Figure 4a). *Mychonastes* was clearly associated with the freshwater reaches of the estuary (Figure 2c and Figure S2a), resulting in negative correlation with salinity (Figure 4a,b, *r* = −.83, *p* < .001). In contrast, *Minidiscus* was more dominant further downstream (Figure 2c and Figure S2a) and hence associated with higher salinity and higher PO_4_ values (Figure 4a,b, salinity: *r* = .77, *p* < .001, PO_4_: *r* = .58, *p* = .010).

## 4 DISCUSSION

### 4.1 Picophytoplankton dominate phytoplankton communities year‐round

We used flow cytometry to quantify picophytoplankton along the Elbe estuary and across seasons and combined the results with composition data obtained from metabarcoding. Our results indicate that picophytoplankton—and therein picoeukaryotes—were the dominant groups of phytoplankton in the Elbe estuary with respect to abundance in the vast majority of the samples. Notably, different picoeukaryote taxa (precisely *Minidiscus* and *Mychonastes*) could each contribute up to 17% to the eukaryotic phytoplankton reads, implying that this group was also relevant in terms of biovolume (see also Martens, Russnak, et al. (2024)) and (Figure 1b). Considering their ubiquitous appearance throughout water bodies around the world (Coello‐Camba & Agustí, 2021; Purcell‐Meyerink et al., 2017; Sathicq et al., 2020; Takasu et al., 2023), it is not surprising that picophytoplankton also play an important role in the Elbe estuary, even though empirical evidence has so far been scarce for this ecosystem. *Mychonastes* in particular has been found in various freshwater bodies (Shi et al., 2018; Yang et al., 2021, 2022; Zhao et al., 2024), and *Minidiscus* is specifically known from marine and brackish habitats (Fernandes & Correr‐Da‐Silva, 2020; Leblanc et al., 2018; Park et al., 2017).

### 4.2 Picophytoplankton abundance follows distinct seasonal and spatial patterns

The peak in picophytoplankton abundance in spring, summer, and fall was likely associated with the elevated temperatures in these seasons (Figures 2a, 3a and 4). Picocyanobacteria were in particular associated with extreme water temperatures (e.g., up to 22°C), and this relationship has been observed in various studies before (e.g., Alegria Zufia et al., 2021; Li et al., 2024). However, other factors not measured in this study, such as sunlight availability and grazing as a factor to terminate blooms, may play an equally important role in shaping seasonal picophytoplankton patterns.

High picophytoplankton abundances at the uppermost station at 609 km (Figure 2a) derive from the inputs of riverine phytoplankton and their growth in the relatively undisturbed area upstream of the city center of Hamburg. A drop in phytoplankton abundance from 609 km toward downstream of Hamburg is a well‐known phenomenon from the area. This is partially explained by local grazing effects (Schöl et al., 2009) but may also be affected by, for example, sinking in the current‐calmed harbor basins (Wolfstein, 1996). Picophytoplankton abundance followed this pattern in our study and hence seems to be affected by these factors. An increase in picophytoplankton abundance in the vicinity of the North Sea (713 km) in summer 2022 may be explained by coastal inputs, especially as salinity was relatively high in this area and season (ca. 20 PSU).

### 4.3 Picophytoplankton are relatively important at extreme temperatures and low light availability

The proportions of picophytoplankton within the phytoplankton communities (Figures 2b and 3b) show the *relative* importance of this size group and can indicate where and when picophytoplankton grow better or get removed less quickly than larger phytoplankton. Picophytoplankton contributions to the phytoplankton communities indicate that picophytoplankton play a major role under extreme environmental conditions with respect to temperature and light availability. The proportions of picocyanobacteria within the phytoplankton communities were highest at high temperatures (e.g., 22°C) following the patterns of their abundance (Figure 2a,b). In contrast, though picoeukaryotes were positively correlated with temperature in terms of cell counts (Figure 4b), and appeared most abundant at 609 km (Figure 2a), their relative importance within the phytoplankton communities was highest at a combination of low temperature and low light availability (e.g., due to turbidity, low sunlight availability in winter). This derived from low contributions in summer in the seasonal dataset (Figure 3b), as well as high contributions in winter 2022 (low temperature, high turbidity) and low contributions in summer 2021 (high temperature) in the spatial dataset and generally higher contributions in the mid estuary (633–692 km) where turbidity was overall higher (Figures 2b and 4a). While further research is needed to disentangle the effects of temperature and turbidity, a positive relationship with turbidity has been observed before (e.g., in Somogyi et al., 2017). Picophytoplankton might have specific strategies in light harvesting (Coe et al., 2021; Liu et al., 2020; Somogyi et al., 2017, 2022; Soulier et al., 2022). Additionally, we found that picoeukaryotes from the Elbe estuary were particularly skilled in utilizing organic compounds (Martens, Ehlert, et al., 2024). Making use of available organic resources such as amino acids and carbohydrates can be an efficient strategy of phytoplankton to deal with—partially very variable—resource availability and provide a steady supply with nutrients (e.g., P, N), more complex substrates (e.g., amino acids), and energy (see e.g., Muñoz‐Marín et al., 2020; Reinl et al., 2022). However, their higher contributions in the mid estuary might also be partially explained by them being removed less rapidly or distinctively by the lethal factors appearing in the area around Hamburg (e.g., grazing, sinking) (Schöl et al., 2009; Wolfstein, 1996). For instance, small picophytoplankton cells can have a reduced sinking velocity compared to larger‐celled phytoplankton. Moreover, while one of the key zooplankton taxa—*Eurytemora* (Schöl et al., 2009)—may utilize picophytoplankton, for example, as part of aggregates (Modéran et al., 2012; Wilson & Steinberg, 2010), they likely prefer to consume larger‐celled phytoplankton, and hence, picophytoplankton might be eliminated less quickly by grazing. Lastly, the positive relationship of the contributions of different picophytoplankton groups with nutrients (NO_3_ and PO_4_) implies that those groups may benefit from high nutrient availability, for instance, in seasons (e.g., winter) and areas (e.g., the mid to lower estuary, 633–713) with overall low phytoplankton concentrations.

### 4.4 Picophytoplankton are not specifically important at the salinity interface

Picophytoplankton have been found to be important at extreme and highly variable salinities, for example, in hypersaline lakes and in the Black Sea (Belkinova et al., 2021; Somogyi et al., 2022) and at intermediate salinities (e.g., 5–10 PSU) in estuaries (Wetz et al., 2011), which are somewhat extreme for both freshwater and saltwater inhabitants. However, our data so far imply that picophytoplankton were overall more abundant and dominant in freshwater and rather high salinity (approx. 20 PSU) likely due to coastal inputs. Nevertheless, some picophytoplankton taxa, such as certain genotypes of *Minidiscus* (see also Martens, Russnak, et al., 2024) as well as *Ostreococcus*, *Bathycoccus*, and *Picochlorum*, which were particularly associated with intermediate salinities (approx. 1–10 PSU), have been associated with brackish habitats (e.g., in Hu et al., 2016; Tragin & Vaulot, 2019) and high salinity tolerances (Foflonker et al., 2016; Somogyi et al., 2022) before. Those groups might fulfill significant ecological functions at the salinity interface of the estuary, for example, as primary producers and as food items for higher trophic levels, the latter regardless of whether they are in particularly good condition.

### 4.5 Taxa composition requires further investigations

In our dataset, *Mychonastes* and *Minidiscus* were the dominant picoeukaryote taxa based on 18S sequencing, with *Nannochloropsis* playing a role in the colder seasons (Figure 2c) and *Synechococcus* and *Microcystis* played a role as picocyanobacteria based on 16S sequencing. The results of the 16S sequencing were specifically limited as sufficient numbers of reads were often not obtained, and we can mostly conclude that these taxa play a role but not further delve into quantitative analysis. However, also the eukaryotic picophytoplankton data are limited, as 18S sequencing (Martens, Russnak, et al., 2024) generated a lot of phytoplankton reads that could not be assigned to a specific genus. Those reads might partially belong to the group of picoeukaryotes and hence the composition of picoeukaryotes might be more complex than shown in our data (Figure 2c and Figure S2a). Moreover, in 2021, metabarcoding was not carried out at 651–665 km, and hence we miss information about this area which was particularly interesting in the flow cytometric results (e.g., with respect to elevated picoeukaryote contributions, Figure 2b). From our data, we can say that *Minidiscus* and *Mychonastes* are likely very important picoeukaryotes in the Elbe estuary. *Minidiscus* was more important from the mid to lower estuary and at elevated salinities (Figures 2c and 4 and Figure S2a) which fits the general distribution of this taxon along brackish and marine habitats (Fernandes & Correr‐Da‐Silva, 2020; Leblanc et al., 2018; Park et al., 2017). In contrast, *Mychonastes*—appearing further upstream—is a genus well known from various freshwater ecosystems (Shi et al., 2018; Yang et al., 2021, 2022; Zhao et al., 2024). Both taxa were more dominant in summer and spring (Figure 2c and Figure S2a), though a positive relationship with temperature was not significant in our data (Figure 4b, see also Martens, Russnak, et al., 2024). Various studies imply that *Mychonastes* can be important throughout different seasons (Shi et al., 2018; Yang et al., 2021, 2022; Zhao et al., 2024), so its higher contributions in summer and spring might be specific for the biome of our study area. Notably, some of the factors mentioned further above that might be beneficial for picophytoplankton—especially in extreme environments—also apply for *Mychonastes*. For instance, concerning light‐harvesting strategies, *Mychonastes* has been found to increase their chlorophyll a content and adjust their pigment composition to varying light availability, and *Mychonastes* appears tolerant of a broad range of light availabilities, which may help them survive under the fluctuating light conditions in the Elbe estuary (Malinsky‐Rushansky, 2002). Additionally, in our former study (Martens, Ehlert, et al., 2024), we found that different strains of *Mychonastes* from the Elbe estuary had a high mixotrophic ability and a flexible strategy to acquire energy and nutrients may add in establishing dominance in our highly variable study area. Unfortunately, there are still few ecological data about *Minidiscus* to compare with. Yet, as a pico‐diatom, *Minidiscus* plays a largely unique ecological role as it does not only benefit from its small size (e.g., with respect to a high surface to volume ratio) but also has a protective silica frustule that may prevent rapid removal by grazing and allow this taxon to appear more dominant in the lower and mid estuary.

### 4.6 Ecological significance and outlook

Methodological limitations—such as an underrepresentation of samples with intermediate to higher salinities—might have affected some of our interpretations; however, consistent findings across a high number of samples included, for example, with respect to the picophytoplankton dominance in terms of cell counts, make it inevitable to conclude that picophytoplankton play a key role in the Elbe estuary. Their high contributions under extreme conditions—for example, high temperatures and low light availability—imply that they occupy ecological niches where larger phytoplankton might struggle to maintain primary production. Here they supply the higher trophic levels—such as micrograzers, filter feeders, and nauplii larvae (see, e.g., Bemal & Anil, 2019; Richard et al., 2022) with energy and essential nutrients and hence maintain the food webs. Beyond, by maintaining primary production, picophytoplankton contribute to the upkeep of the biological pump (Basu & Mackey, 2018), that is, the transfer of carbon from (atmospheric) CO_2_ toward aquatic biomass and finally carbon sequestration. However, due to their advantage at higher temperatures, (pico) cyanobacteria may become more dominant in the Elbe estuary under global warming (see also Flombaum & Martiny, 2021), which might affect food webs due to the relatively low nutritional value and possible toxicity of cyanobacteria (Ger et al., 2016; Sim et al., 2023). Our results emphasize the importance to include the so far underrated group of picophytoplankton in (estuarine) research and provide insights into the comparability of techniques (e.g., flow cytometry, metabarcoding) for detecting (pico) phytoplankton communities.

## CONFLICT OF INTEREST STATEMENT

We declare we have no competing interests.

## Supporting information


Data S1:

Data S2:

## Data Availability

Flow cytometric and metabarcoding data are provided in the supplementary material (Table S5). The metabarcoding raw data will be published on ENA. Flow cytometric raw data are available on request from the authors.
