# Supplementary material for: Picophytoplankton prevail year‐round in the Elbe estuary
Source: Plant Environ Interact. 2024 Oct 27;5(5):e70014. doi: 10.1002/pei3.70014 (PMC11513158; doi:10.1002/pei3.70014)
Supplement: Supplementary file 1 — Data S1: [file PEI3-5-e70014-s001.pdf]

# Supplementary figures

## *Picophytoplankton prevail year-round in the Elbe estuary*

Nele Martens[1], Johanna Biederbick[1] and C.-Elisa Schaum[1,2]

Affiliations:

[1] Institute of Marine Ecosystem and Fishery Science, Olbersweg 24, 22767 Hamburg, Germany
[2] Center for Earth System Research and Sustainability, Bundesstraße 53-55, 20146 Hamburg, Germany

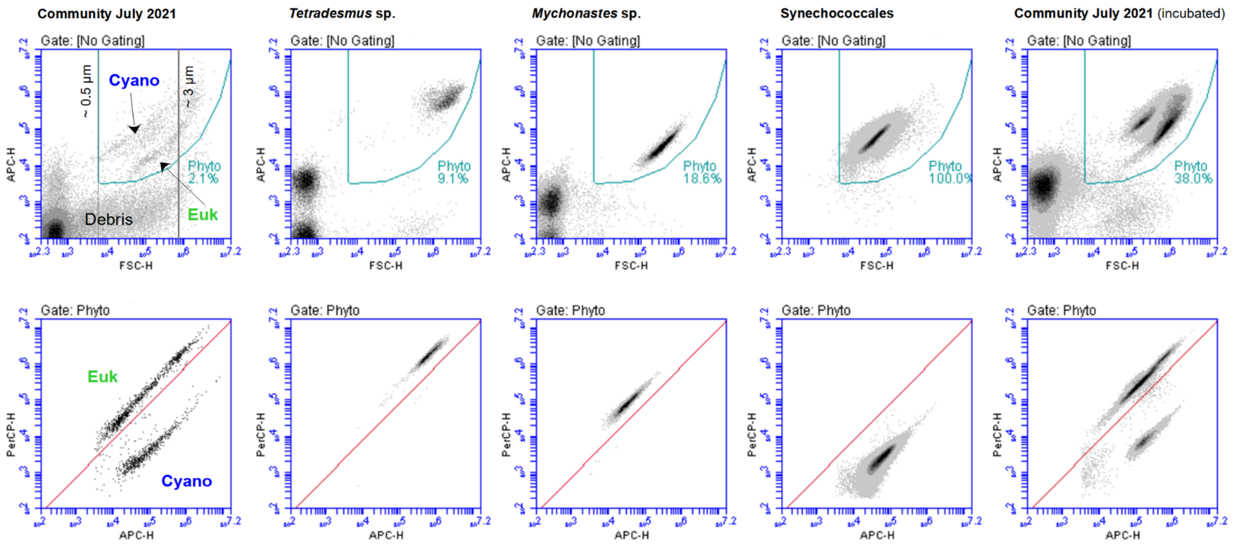


**Fig. S1: Cytogram examples of communities and single strains of phytoplankton isolated from the Elbe estuary.** We used approximately 40 different samples (isolated taxa and laboratory incubated communities) from the Elbe estuary to determine the borders between phytoplankton and other suspended matter (e.g. bacteria, debris) and between eukaryotes and phycocyanin-rich cyanobacteria based on the flow cytometric properties. The gating of phytoplankton (phyto; upper panels) was based on forward scatter (FSC ; ~ size) and APC (allophycocyanin fluorescence; red fluorescence, filter 675 nm, laser 640 nm). Here we also set a lower size border of around 0.5 µm which was proxied in between the FSC values of 0.12 and 1 µm beads. The gating of eukaryotes (euk) and cyanobacteria (cyano) (lower panels) was done based on the ratio of APC and PerCP (peridinin-chlorophyll-protein complex fluorescence; red fluorescence, filter 670 nm, laser 488 nm). Lastly, we defined the picophytoplankton threshold based on 3 µm beads.

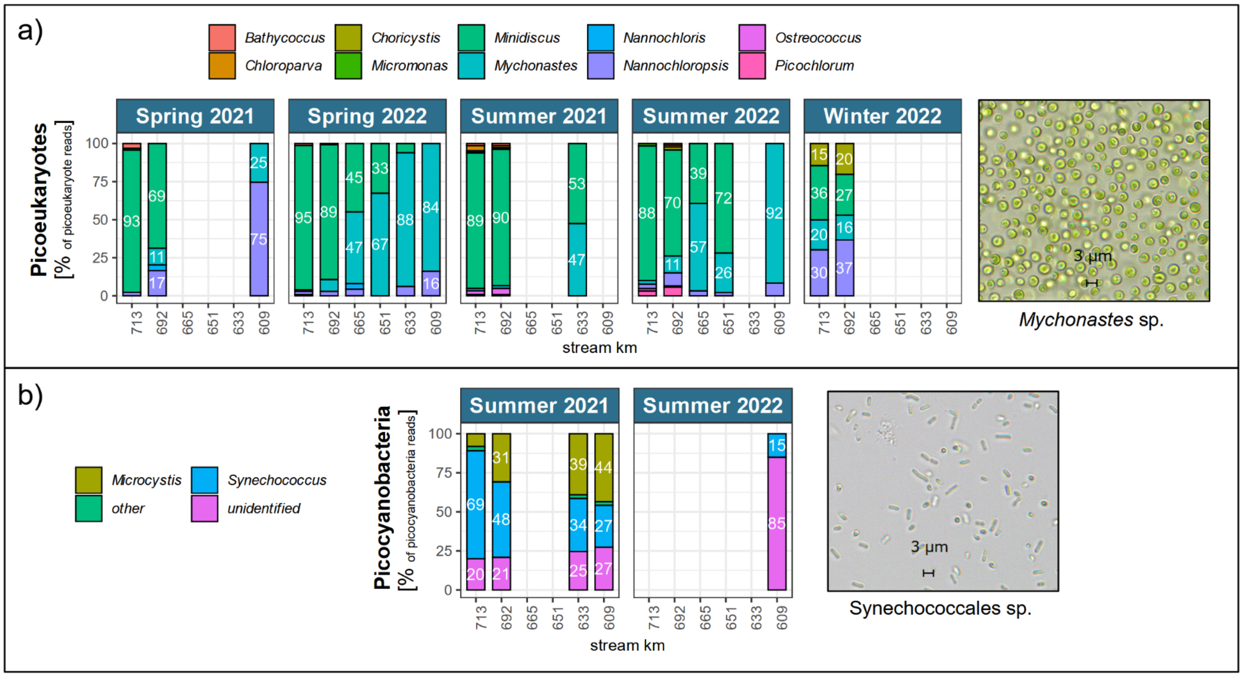


**Fig. S2: Spatial contributions of picoeukaryote genera to picoeukaryote 18S rRNA reads (a) and picocyanobacteria genera to picocyanobacteria 16S rRNA reads (b).** Labels are shown where contribution was at least 10 %. Station is used as a factor (i.e. bars have the same distance independent of the actual location). Note that in each a) and b) we selected different taxa that *can* be or are *usually* understood as picophytoplankton (< 3 µm). Cells of these taxa however *can* sometimes be larger than 3 µm or form colonies and some unidentified cyanobacteria might not be < 3 µm. Moreover, the number of reads can depend on cell size (Godhe et al., 2008). Hence, the selection in this plot does not necessarily accurately represent the picophytoplankton obtained from flow cytometry, where the definition is based on their actual cell size and abundance (see also fig. 1b). It should also be considered that metabarcoding includes phytoplankton that could not be identified to genus level and hence does not appear as they cannot be assigned to the size group of picophytoplankton. Where bars are missing - or in case of picocyanobacteria even complete panels - metabarcoding was either not carried out (651 - 665 km in 2021) or data was removed due to low number of picocyanobacteria or picoeukaryotes reads (< 100) (all other cases). Photos show examples of picophytoplankton strains isolated from the Elbe estuary (Martens et al., 2024a).

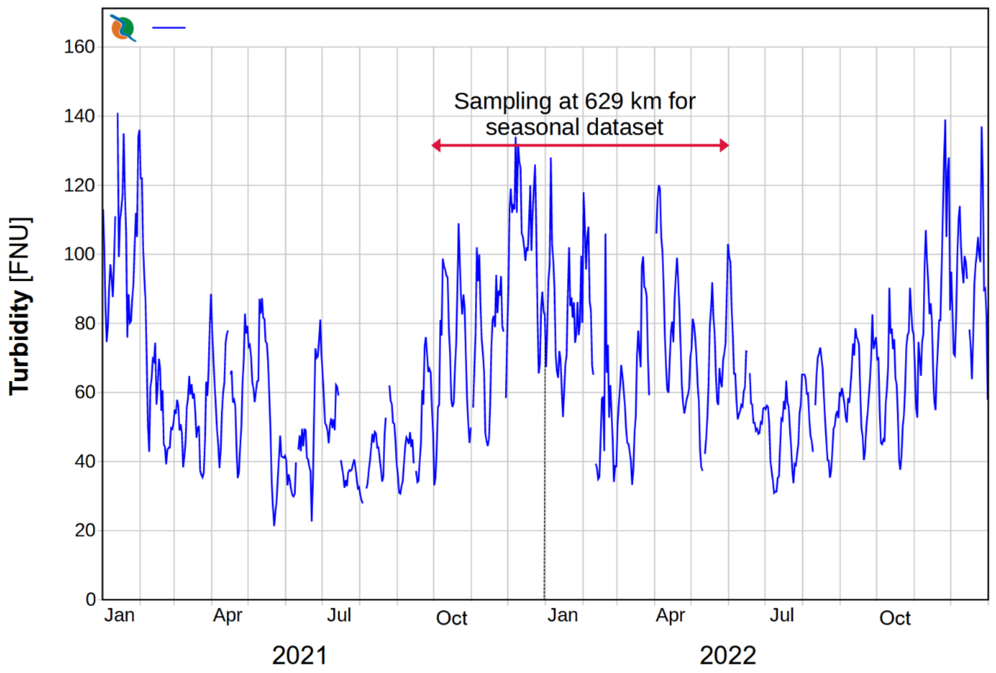


**Fig. S3: Average daily turbidity (FNU) at the station Seemannshöft (approx. 629 km) in the Elbe estuary based on data from the FGG Elbe database (FGG Elbe, 2024) from 2021 - 2022.** Figure was obtained directly from the FGG database, but modified by removing German texts.
